# Programmed death-ligand 1 expression by digital image analysis advances thyroid cancer diagnosis among encapsulated follicular lesions

**DOI:** 10.18632/oncotarget.24833

**Published:** 2018-04-13

**Authors:** Anne M.-Y. Hsieh, Olena Polyakova, Guodong Fu, Ronald S. Chazen, Christina MacMillan, Ian J. Witterick, Ranju Ralhan, Paul G. Walfish

**Affiliations:** ^1^ Alex and Simona Shnaider Research Laboratory in Molecular Oncology, Sinai Health System, Toronto, ON, Canada; ^2^ Joseph and Mildred Sonshine Family Centre for Head and Neck Diseases, Sinai Health System, Toronto, Ontario, Canada; ^3^ Department of Pathology and Laboratory Medicine, Sinai Health System, Toronto, Ontario, Canada; ^4^ Laboratory Medicine and Pathobiology, University of Toronto, Toronto, Ontario, Canada; ^5^ Department of Otolaryngology-Head and Neck Surgery, Sinai Health System, Toronto, Ontario, Canada; ^6^ Department of Medicine, Endocrine Division, Sinai Health System and University of Toronto Medical School, Toronto, Ontario, Canada

**Keywords:** thyroid cancer, digital image analysis, programmed death-ligand 1, invasion, protein biomarker

## Abstract

Recognition of noninvasive follicular thyroid neoplasms with papillary-like nuclear features (NIFTP) that distinguishes them from invasive malignant encapsulated follicular variant of papillary thyroid carcinoma (EFVPTC) can prevent overtreatment of NIFTP patients. We and others have previously reported that programmed death-ligand 1 (PD-L1) is a useful biomarker in thyroid tumors; however, all reports to date have relied on manual scoring that is time consuming as well as subject to individual bias. Consequently, we developed a digital image analysis (DIA) protocol for cytoplasmic and membranous stain quantitation (ThyApp) and evaluated three tumor sampling methods [Systemic Uniform Random Sampling, hotspot nucleus, and hotspot nucleus/3,3′-Diaminobenzidine (DAB)]. A patient cohort of 153 cases consisting of 48 NIFTP, 44 EFVPTC, 26 benign nodules and 35 encapsulated follicular lesions/neoplasms with lymphocytic thyroiditis (LT) was studied. ThyApp quantitation of PD-L1 expression revealed a significant difference between invasive EFVPTC and NIFTP; but none between NIFTP and benign nodules. ThyApp integrated with hotspot nucleus tumor sampling method demonstrated to be most clinically relevant, consumed least processing time, and eliminated interobserver variance. In conclusion, the fully automatic DIA algorithm developed using a histomorphological approach objectively quantitated PD-L1 expression in encapsulated thyroid neoplasms and outperformed manual scoring in reproducibility and higher efficiency.

## INTRODUCTION

The diagnosis of the borderline lesions are challenging in clinical practice [1–3]. The identification of highly indolent noninvasive follicular thyroid neoplasms with papillary-like nuclear features (NIFTP) from the invasive malignant encapsulated follicular variant of papillary thyroid carcinoma (EFVPTC) can prevent overdiagnosis and overtreatment of NIFTP patients [4]. There is increasing recognition to utilize biomarker analysis to complement histopathological diagnosis and prognosis management decisions. Our laboratory has been investigating protein biomarkers for thyroid carcinomas [5–10] and showed that programmed death-ligand 1 (PD-L1) overexpression, predominantly localized in cytoplasm and occasionally in plasma membrane of tumor cells, is a useful prognostic marker for aggressive papillary thyroid cancer and its variants [11]. Shi *et al.* independently confirmed the positive correlation of increased PD-L1 expression with PTC recurrence [12]. Fu *et al.* in our lab recently demonstrated PD-L1 immunohistochemistry (IHC) staining as an ancillary technique in distinguishing invasive EFVPTC from NIFTP and supported the reclassification of NIFTP [13, 14] even if the tumor size is more than 4 cm or the patients are older than 45 years of age [15]. However, all reports to date have relied on manual scoring that is time consuming and can be subject to individual bias. As pathology evolves into a more digital discipline, the need for the development of a digital image analysis (DIA) tool that could provide an efficient, reproducible, and accurate quantitation of protein biomarker expression came into focus.

Conventional DIA IHC stain quantitation requires defining an area surrounding each nucleus as cytoplasm from the nucleus border [16–21]. With this bottom-up approach wherein there is a sequential progression from the initial segmentation of the target cell nucleus to the derivation of the cytoplasmic space, correct identification and proper delineation of viable follicular cell nuclei are prerequisite to the quality of the extracted data. Tumor cell phenotyping is challenging without a definitive cell marker. The difficulties are twofold: (i) differentiating target nuclei in a multicellular tissue where different cells have overlapping morphometric characteristics requires a panel of criteria (i.e. size, perimeter, texture, and immunohistochemistry biomarker [22–26]) and fine tuning techniques (i.e. pattern recognition that utilizes correlation to neighbor nuclei features [27]); and (ii) there is a large variation in nuclear morphometry among cancer variants of the same tissue and the progression of disease [28–32]. An alternative top-down DIA approach may circumvent the above mentioned challenges. The protocol starts by first exploiting tissue components and structures. The tissue histomorphology outlined then breaks down into smaller segments, where the region of interest (ROI) for IHC quantitation is specified. The latter approach more closely mimics the pathologists’ practice in assessing tissue staining. Prior knowledge of histomorphology and staining pattern is activated to define tissue components, following which the target cells and area of interest for IHC quantification are derived.

High efficiency DIA can be achieved through strategically sampling a tumor. For pixel-precision in feature extraction, DIA quantification is usually conducted at high resolution (20X). Analyzing the whole lesion at high resolution is time consuming, whereas analyzing at low resolution reduces accuracy. One approach to circumvent this problem is to screen the tumor at low resolution to extract a sampling subset and then within these subROIs to perform DIA quantitation at high resolution. In the initial semi-manual DIA, the user defines subROIs in the tumor where the DIA protocol package (ThyApp), a systematic image processing operation algorithm developed in our lab using the Visiopharm platform, is applied. Moving towards minimizing inter- and intra-observer variability, automatic subROI selection methods are applied. We evaluated tumor sampling methods including uniform systematic random sampling (SURS) and a combination of nuclear density and 3,3′-Diaminobenzidine (DAB) intensity hotspots. The aim of this study was to develop a fully automatic DIA algorithm that provides an efficient, objective, and accurate quantitation of PD-L1 expression. A standardized technical and interpretational method can be used as a useful aid to histopathological diagnosis to effectively distinguish NIFTP from invasive EFVPTC and avoid overdiagnosis and overtreatment of NIFTP.

## RESULTS

### Clinical and pathological characteristics of the patient cohort

The clinicopathological characteristics of the study cohort are summarized in Table 1. Among the 153 cases meeting the inclusion criteria, 26 cases were classified as benign nodules, 48 as NIFTP, 44 as EFVPTC and 35 cases with predominant coexisting lymphocytic and Hashimoto's thyroiditis (LT).

**Table 1:** Demographics and clinicopathologic features of patient cohort

Characteristics	Total cases (*n* = 153)
Age, mean (range)	50.6 (15–79)
**Gender**	***n* (%)**
Male	
<45 years	10 (7)
≥45 years	22 (14)
Female	
<45 years	47 (31)
≥45 years	74 (48)
**Pathological diagnosis**	***n* (%)**
Benign	
Multiple hyperplastic nodules	23 (15)
Cyst	1 (0.7)
Follicular adenoma	2 (1.3)
Neoplasm	
NIFTP	48 (31)
EFVPTC	44 (29)
Lymphocytic Thyroiditis (LT)^*^	35 (23)

^*^Lymphocytic thyroiditis (LT) was separated from other categories because of coexisting advanced Lymphocytic or Hashimoto's thyroiditis.

Noninvasive follicular thyroid neoplasms with papillary-like nuclear features (NIFTP); encapsulated follicular variant of papillary thyroid carcinoma (EFVPTC).

### DIA of PD-L1 expression distinguished between NIFTP and invasive EFVPTC

**Figure 1:**
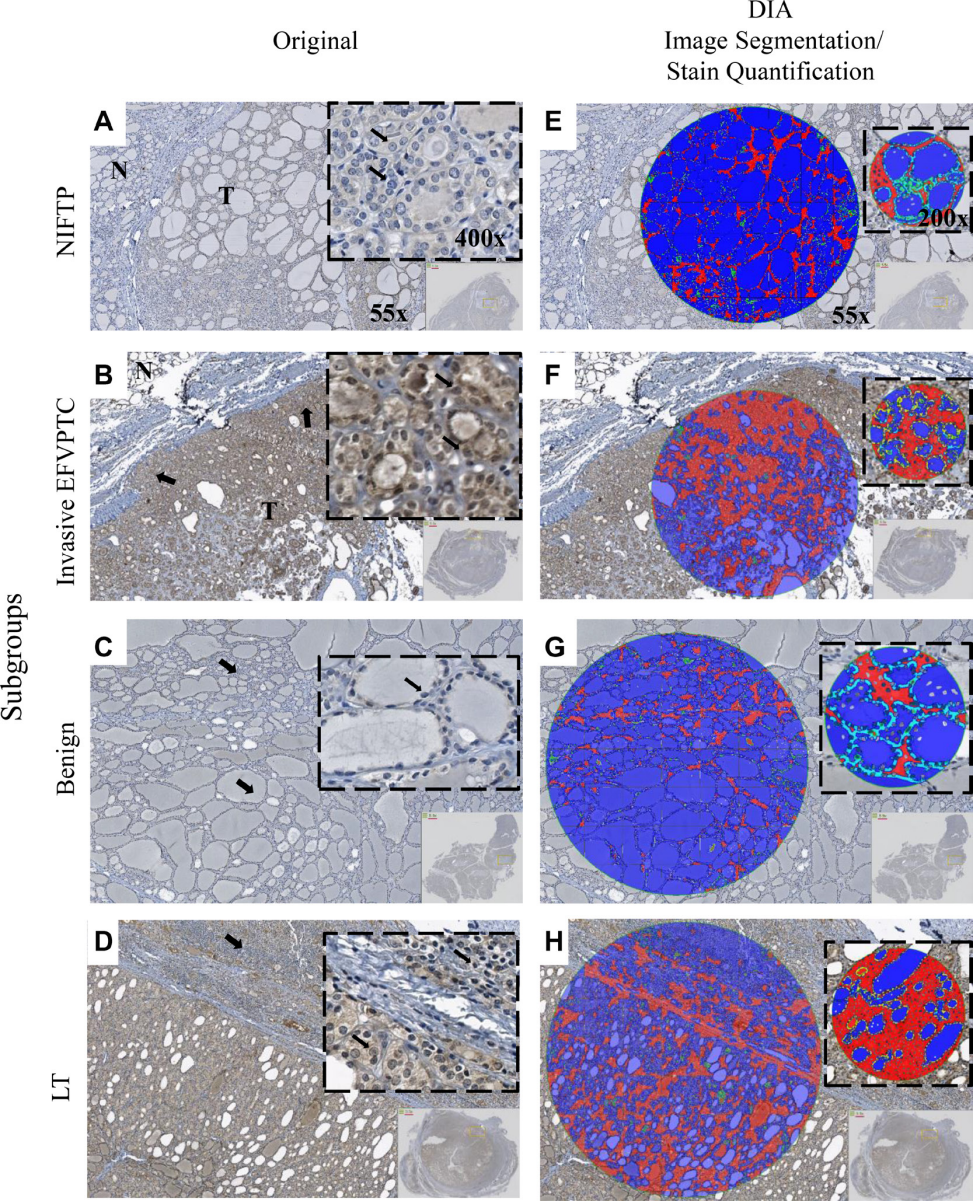
Immunohistochemistry staining and digital image analysis of PD-L1 expression in NIFTP, invasive EFVPTC, Benign and LT Original bright field photomicrographs are shown in the first column. Tissue sections of: (**A**) NIFTP showed follicular architecture. Inset: PTC cytological features including nuclear enlargement, grooves, micronuclei, pale to clear chromatin and crowding (thin black arrows). Faint PD-L1 expression was detected in NIFTP; (**B**) invasive EFVPTC showed the focal capsular invasion (thick black arrows) and the follicular growth pattern of the tumor (T) as compared to normal thyroid (N). Inset: typical PTC features including enlarged nuclei with central clearing, grooves and crowding (thin black arrows). Moderate to strong PD-L1 expression was observed in EFVPTC with invasion; (**C**) benign hyperplastic nodules showed follicular of various sizes (thick black arrows). Inset: uniform round nuclei (thin black arrows). Minimal detectable PD-L1 expression was observed in benign nodules; and (**D**) LT showed severe lymphoplasmacytic infiltration with germinal center formation and follicles (thick black arrows). Inset: extensive lymphocytic and plasmacytic infiltrate with oxyphilic metaplasia of follicular cells (thin black arrows). Mild to moderate PD-L1 expression was observed. (**E**–**H**) Layer images showed DIA segmentation including parafollicular space (red) and colloid (blue). Zoomed in images shown in the insets, DIA quantified DAB and pseudocolored PD-L1^+^ (yellow) and PD-L1^-^ (light blue) in the follicular cell. Image magnification are ×55 for (A–H), ×400 for (A–D) insets and ×200 for (E–H) insets, respectively. Capsule tissue in Figure 1 was not considered for DIA stain quantitation. Programmed-death ligand 1 (PD-L1); noninvasive follicular thyroid neoplasms with papillary-like nuclear features (NIFTP); encapsulated follicular variant of papillary thyroid carcinoma (EFVPTC); encapsulated follicular lesions/neoplasms with lymphocytic or Hashimoto’s thyroiditis (LT); papillary thyroid cancer (PTC); digital image analysis (DIA); 3,3′-Diaminobenzidine (DAB)

**Figure 2:**
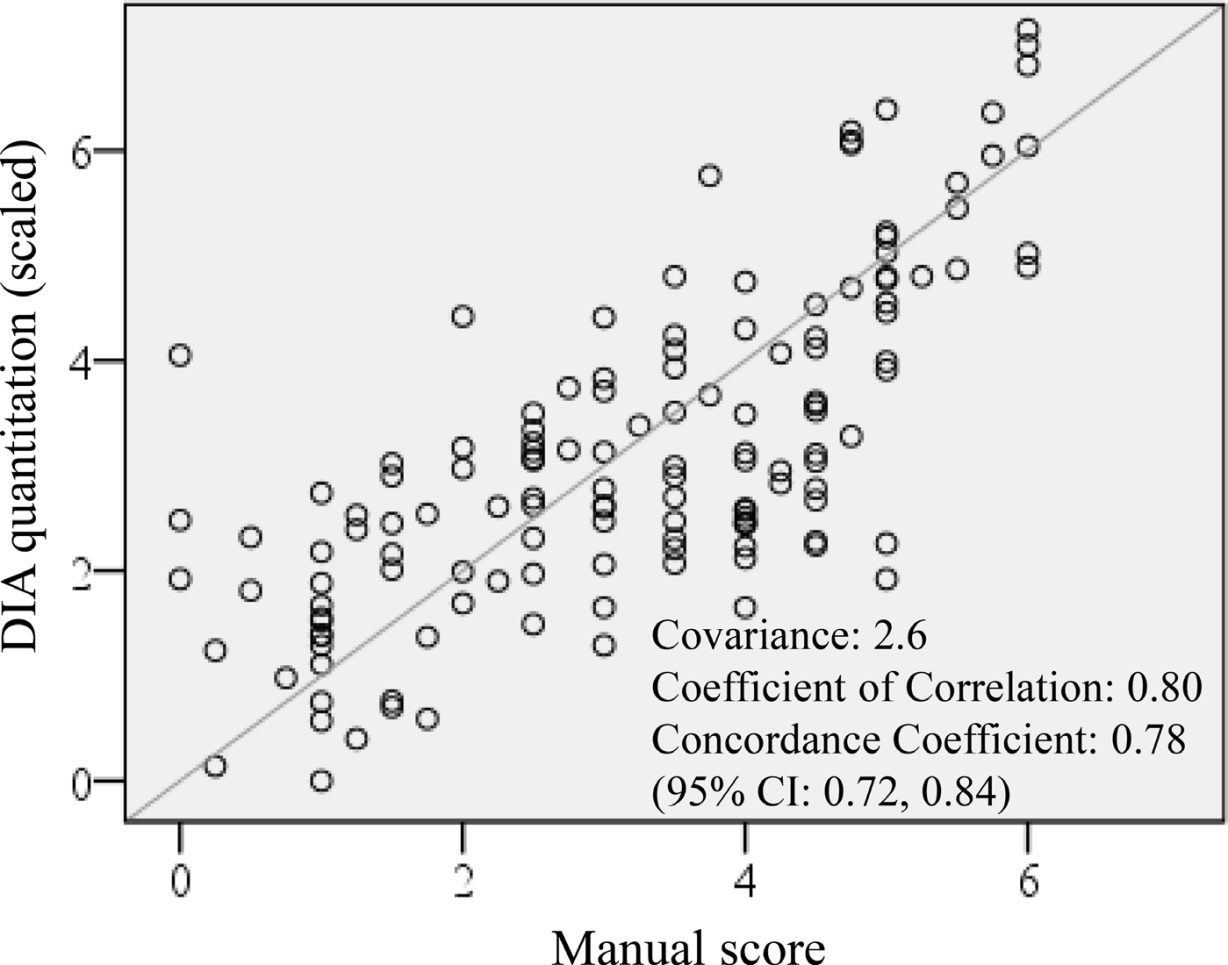
DIA PD-L1 expression quantitation concordance with manual scoring DIA quantitation showed high correlation and substantial correlation coefficient with manual scoring, *R* = 0.80 and Rc = 0.78, respectively. Digital image analysis (DIA); Programmed death-ligand 1 (PD-L1); correlation (R); Concordance correlation coefficient (Rc).

With the reclassification of NIFTP and the emergence of PD-L1 as a protein biomarker to differentiate NIFTP from invasive EFVPTC, we examined whether the DIA application protocol developed, ThyApp, could be used to assess PD-L1 expression and to distinguish the subgroups. Representative images for each subgroup are shown in Figure 1. From the original bright field images, it was observed DAB intensities were elevated in invasive EFVPTC and lymphocytic thyroiditis (LT) in contrast to NIFTP and benign nodules (Figure 1B, 1D versus 1A, 1C). The ThyApp segmented the tissue into its components and specified the follicular cells accordingly (Figure 1E–1H). Stain mean intensity and percent positivity were quantitated (shown in yellow or light blue as PD-L1^+^ or PD-L1^-^ area respectively, Figure 1E–1H insets). Controls for immunohistochemistry including oral cancer and anaplastic thyroid cancer for positive tissue controls, and encapsulated FVPTC for negative and positive controls are shown in Supplementary Figure 1A–1D, respectively. One-way ANOVA of DIA quantitated parameters including percent positivity and DAB mean intensity in Table 2 revealed PD-L1 expression significantly increased in invasive EFVPTC as compared to NIFTP (*p* < 0.001), and to benign nodules (*p* < 0.001); but none was observed between NIFTP and benign nodules (*p* = 0.113). The coexisting predominant lymphocytic/Hashimoto's thyroiditis (LT) enhanced PD-L1 expression in tumor cells and follicular cells within the inflammatory infiltrated area. Therefore, the PD-L1 staining in the presence of increased lymphocytic infiltration needs to be interpreted with caution. Using a similar but not identical patient cohort from a previously published paper [13], DIA PD-L1 expression effectively differentiated NIFTP from invasive EFVPTC. In Figure 2 comparing manual scores and DIA quantitation of individual cases (each open circle represents one case), high correlation and substantial concordance coefficient, 0.80 and 0.78, respectively, were observed.

**Table 2:** Multiple comparisons of DIA analyzed PD-L1 expression

DIA Parameter	Group (I)	Group (J)	Mean Difference (I–J)	Std.Error	Sig. (*p*)	95% CI	
						Lower Bound	Upper Bound
Percent Positivity	NIFTP	EFVPTC	–23^*^	4.9	**<0.001**	–36	–11
		Benign	13	5.7	0.1	–2	28
		LT	–14^*^	5.2	**0.04**	–28	–0.6
Mean Intensity	NIFTP	EFVPTC	30^*^	6.2	**<0.001**	14	46
		Benign	–16	7.3	0.1	–35	2
		LT	19^*^	6.6	**0.02**	2	37

^*^The mean difference is considered significant when *p* < 0.05.

>1000 viable tumor cells per case were evaluated.

Digital image analysis (DIA); programmed death-ligand 1 (PD-L1); noninvasive follicular thyroid neoplasms with papillary-like nuclear features (NIFTP); encapsulated follicular variant of papillary thyroid carcinoma (EFVPTC); encapsulated follicular lesions/neoplasms with lymphocytic or Hashimoto's thyroiditis (LT).

### Thyroid application protocol package development

**Figure 3:**
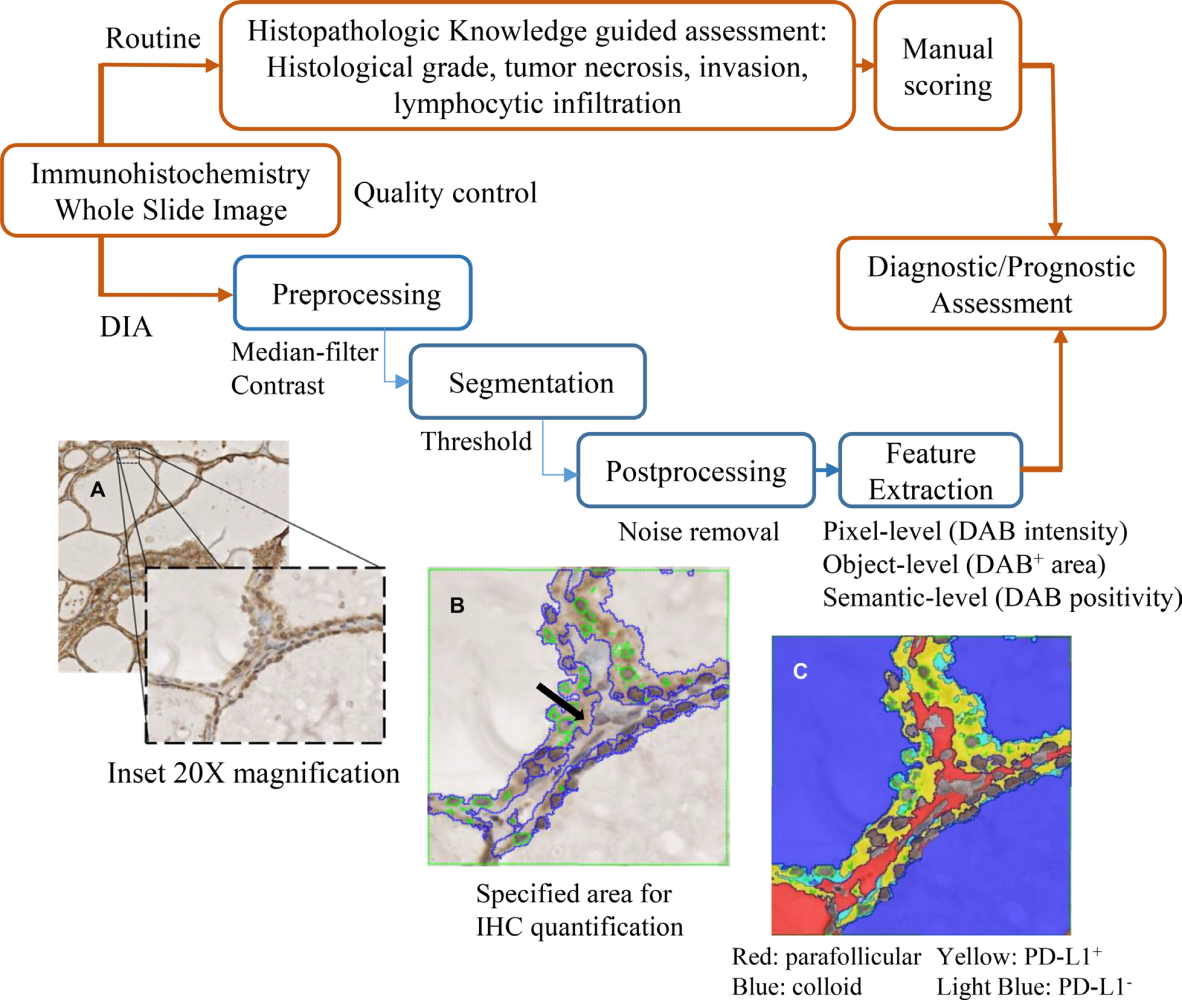
Overview of the DIA pipeline and histomorphological approach The DIA developed in this study (blue/bottom panel) is compared with the histopathology-based assessment (red/top panel). In DIA, the whole slide image was imported to software system and then processed by serial operations (pre-processing, segmentation, and post-processing) in the App. (**A**) In the bright field photomicrographs, higher resolution (×200 magnification) was shown for the DIA close-up view. (**B**) Follicular cell (area delineated by DIA pseudocolor blue lines), excluding the nuclei (area delineated by green lines) were accurately specified (black arrow). (**C**) IHC was quantified in this region and threshold gated into DAB^+^ (yellow) or DAB^-^ (light blue), representing PD-L1^+^ or PD-L1^-^ areas respectively. The Visiopharm integrator system output parameters included DAB intensity and DAB percent positivity. Digital image analysis (DIA); application protocol package (App); Immunohistochemistry (IHC); 3,3′-Diaminobenzidine (DAB); Programmed death-ligand 1 (PD-L1)

Thyroid App was developed to quantify PD-L1 expression in follicular cells. Outlined in Figure 3 illustrated the series of computer-assisted operations performed on scanned IHC stained histology slides. Median-filter was used to remove background noise in the image, and color deconvolution and thresholding were applied to differentiate tissue components. The software subsequently pseudocolor labelled each segmented object. Post-processing further fine-tuned the labelling by morphometry. Activating prior knowledge of the geometrical relationship between the stroma and colloid, a morphological operator selected the interface composed of follicular cells. Nuclei were identified by Hematoxylin filter and excluded from the stain quantitation (Figure 3A and 3B). Features including DAB mean intensity and percent positivity were extracted as DIA output parameters for statistical analysis (Figure 3C).

### Thyroid App verification – robustness to histomorphology variance

**Figure 4:**
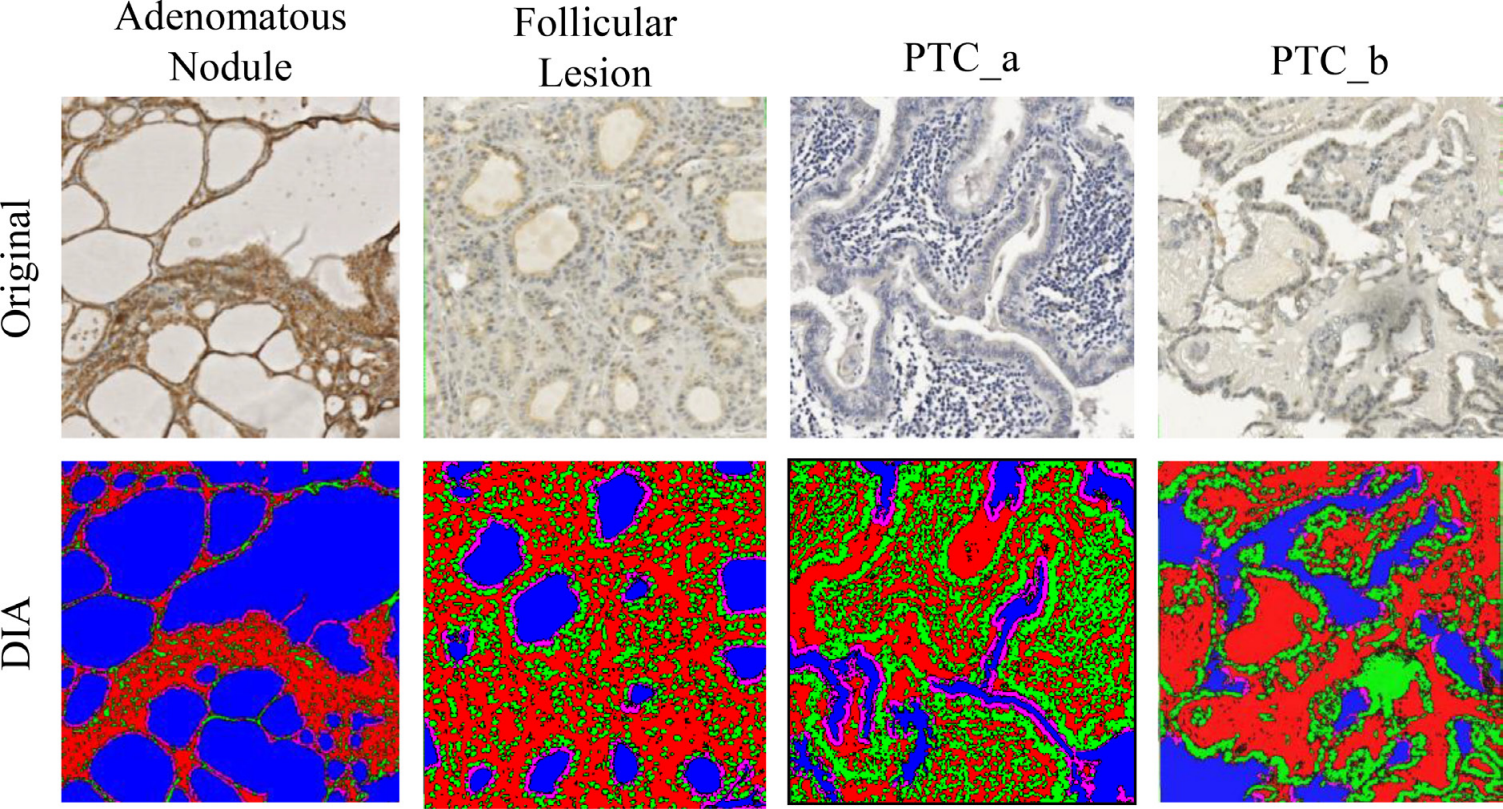
Application of ThyApp to PD-L1 expression analysis in thyroid tissues The developed algorithm using histomorphological approach was used for quantitation of PD-L1 expression on Nanozoomer scanned images representing distinctive diseased thyroid histomorphology shown in the top panel. The robustness of the App was examined based on segmented results shown in the bottom panel with corresponding layer images with color labels; all pixels were sub-classified into colloid (blue), parenchyma (pink), stroma (red), and nucleus (green). Thyroid application protocol package (ThyApp); programmed death-ligand 1 (PD-L1).

The robustness of ThyApp's histomorphological approach to segment thyroid tissue components was examined. DIA was applied to thyroid neoplasms, with a variety of distinctive histomorphologies ranging from adenoma to follicular thyroid carcinoma (FTC), and to typical papillary thyroid carcinoma (PTC), representing the complexity of the surgical specimens analyzed in the clinics. Shown in Figure 4, ThyApp delineated the follicular cells for DIA IHC quantitation in adenomatous nodule, FTC, and two PTCs, demonstrating the algorithm's versatility to quantitate thyroid tissues.

### Thyroid App verification – reflectance of stain variance

**Figure 5:**
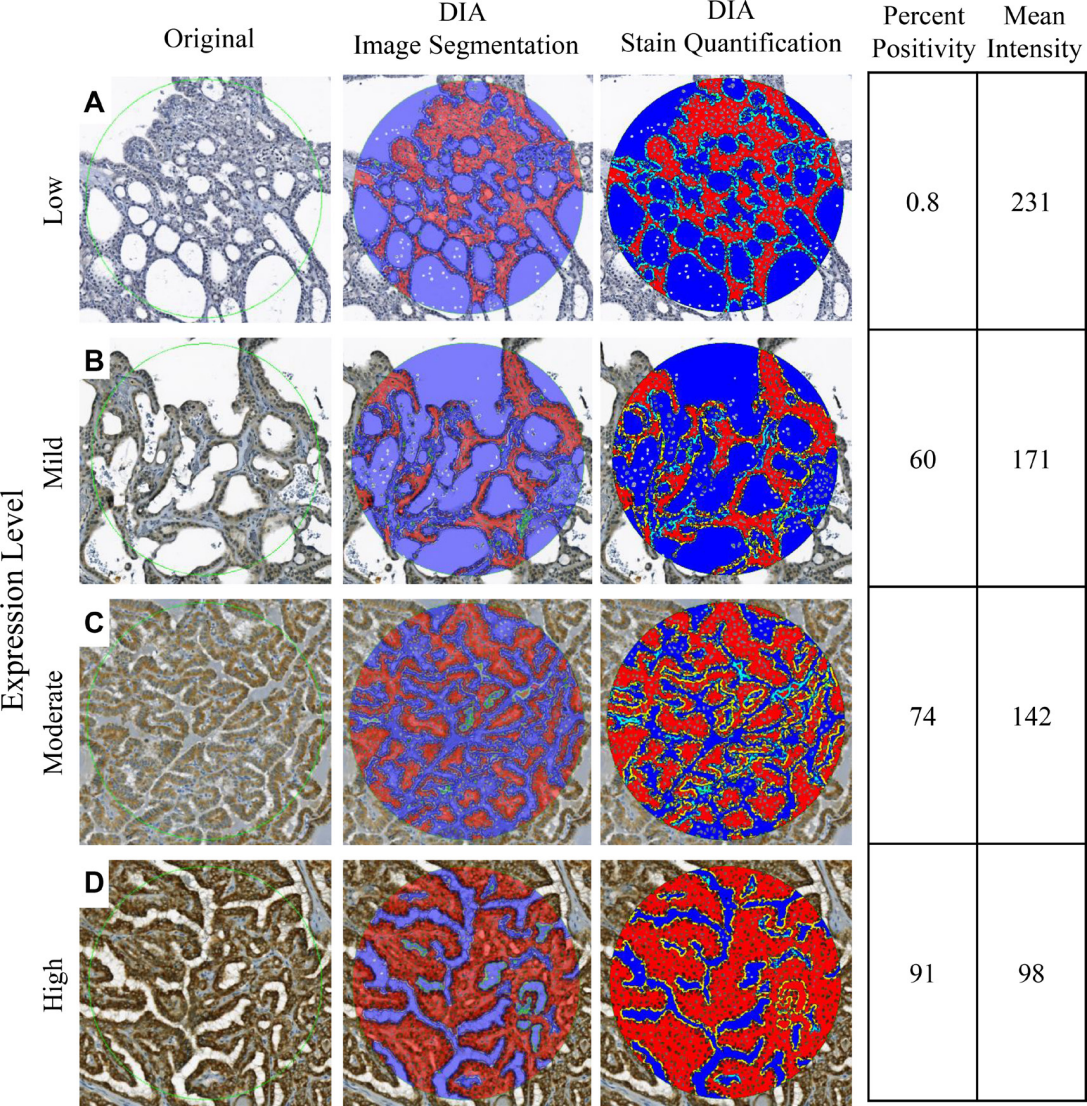
Reflectance of IHC staining Representative digital images of low (**A**), mild (**B**), moderate (**C**), and high (**D**) PD-L1 expressions were digitally analyzed by ThyApp. The segmented results shown by the layer images (middle column, at 50% transparency) accurately labeled thyroid tissues subcomponents. Immunohistochemistry stain positivity and mean intensity was subsequently quantified. With an increase in protein expression level, percent positivity increased from 0.8 to 91%, and mean intensity color values decreased from 231 to 98. Magnification ×200. Immunohistochemistry (IHC); programmed death-ligand 1 (PD-L1); Thyroid application protocol package (ThyApp).

To examine whether the DIA quantitation accurately assessed the IHC staining, photomicrographs of visually apparent low to high DAB intensity were analyzed by ThyApp (Figure 5). The exported DIA parameters, percent positivity and mean intensity, reflected PD-L1 expression level in the images. In the ordinal order of low to high DAB stain intensity, the DAB positive areas increased from 0.8 to 91 %, and DAB mean intensity with color values decreased from 231 to 98 (light to dark DAB from 255 to 0). In a larger DIA quantitated sample size with 113 images each categorized into low, mild, moderate and high PD-L1 expression subgroups according to their manual scores from 0–2, 2.1–4, 4.1–5.9 and 6–7, respectively, ANOVA revealed the average DAB mean intensity in each subgroup significantly differentiated from each other (*p* < 0.001 for all groups, Table 3).

**Table 3:** DIA verification of stain variance

Group (I)	Group (J)	Mean Difference (I–J)	Std.Error	Sig. (*p*)	95% CI	
					Lower Bound	Upper Bound
Low expression	Mild	25^*^	5.4	***p* < 0.001**	11	39
	Moderate	42^*^	5.4	***p* < 0.001**	28	56
	High	83^*^	5.8	***p* < 0.001**	68	98

^*^The mean difference is considered significant when *p* < 0.05.

>1000 viable tumor cells per case were evaluated.

### DIA outperformed manual scoring

In addition to ANOVA analysis that revealed the relationship between PD-L1 expression score mean averages in the subgroups, distribution of the expression scores in each of the subgroups was examined. The scores were from either manual scoring or DIA. With a large sample size, normal distribution of the frequency histogram for individual subgroup could be an indicator for objective assessment and evidence supporting true differentiation of the groups. Normal distribution was observed in all fully automatic DIAed subgroups (except LT), shown by the Shapiro-Wilk normality test with significance above 0.05; whereas, in manual scoring and manual subROI, NIFTP scores failed the normality test (Table 4). Digitally analyzed LT parameter values skewed towards low scores suggesting ThyApp could potentially mitigate the confounding factor from infiltrated lymphocytes through its histomorphological approach. Interobserver (two independent scorers) concordance correlation coefficient (Rc) was only satisfactory at 0.72 (95% CI: 0.65, 0.79) in manual scoring versus 1 in DIA. Moreover, manual scoring of 100 tumor cells took ∼3 min. in contrast to DIA > 1000 follicular cells in less than one minute. In this study, ThyApp outperformed manual scoring providing an objective and reproducible quantitation of PD-L1 expression at higher efficiency.

**Table 4:** Shapiro-Wilk normality test in DIA quantitation versus manual scoring

	Shapiro-Wilk Significance				
	Manual Score	Digital Image Analysis			
		Manual subROI	SURS 3%	Hotspot Nucleus	Hotspot NuDAB
**NIFTP**	**0.005**	**0.006**	0.1	0.1	0.6
**EFVPTC**	0.1	0.8	0.1	0.1	0.1
**Benign**	0.1	0.2	0.5	0.4	0.1
**LT**	0.3	0.2	0.1	**0.03**	0.3

^*^significant when *p* < 0.05.

Digital image analysis (DIA); programmed death-ligand 1 (PD-L1); noninvasive follicular thyroid neoplasms with papillary-like nuclear features (NIFTP); encapsulated follicular variant of papillary thyroid carcinoma (EFVPTC); encapsulated follicular lesions/neoplasms with lymphocytic or Hashimoto's thyroiditis (LT).

### SubROI method evaluation

**Figure 6:**
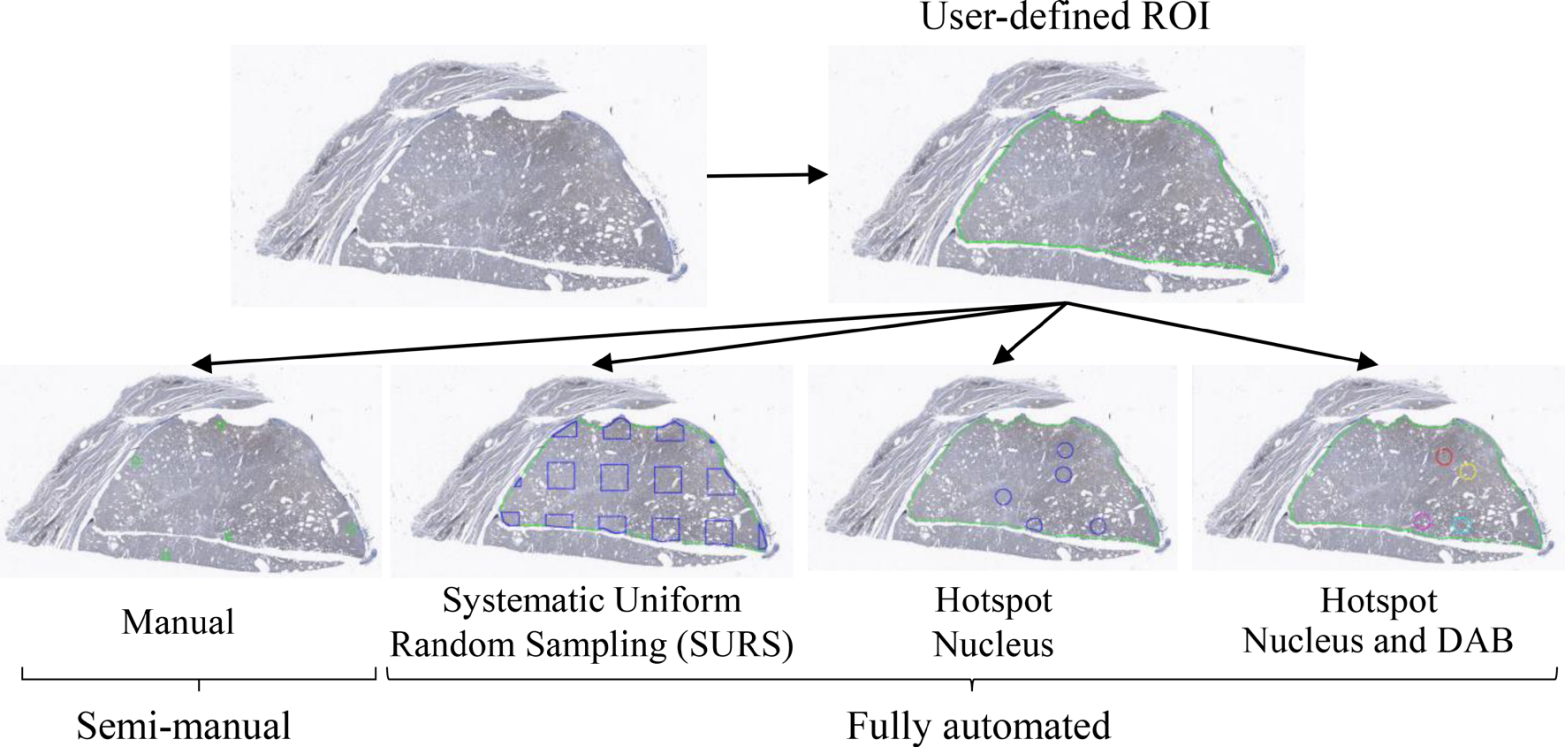
Tumor sampling methods Upon receiving digital whole slide images, an operator delineated the tumor tissue (ROI). Next, areas within the ROI, subROIs, were selected by one semi-manual and three fully automatic subROI methods. In semi-manual, subROIs for DIA were manually selected by a histopathologist. Three fully automatic subROI tumor sampling methods: Systematic Uniform Random Sampling (SURS) (a specified fraction of tumor sampled); hotspot nucleus (Heatmap algorithm selecting subROIs at highest tumor cell nuclei density); hotspot nucleus and DAB (Heatmap algorithm selecting subROIs at highest tumor cell nuclei density and DAB positivity) were implemented and performance evaluated. Region of interest (ROI); digital image analysis (DIA); 3,3′-Diaminobenzidine (DAB).

Moving towards DIA full-automation, upon user-defined tumor ROI, four tumour sampling methods (manual, SURS, Hotspot Nucleus, and Hotspot Nucleus and DAB) in Figure 6 were individually implemented and evaluated. To determine the optimal area fraction required for SURS to represent the whole tumor cross section, a sample fraction study of five PD-L1 stained thyroid tissues was conducted. Three percent tumor area was found to be ideal considering measurement variance and time to process (∼5 min.). Scoring smaller areas induced higher variance and scoring larger areas increased processing time: scoring 5 % took ∼8 min and scoring 10 % took ∼18 min per slide. Hotspot Nucleus and Hotspot Nucleus and DAB analyzing a fixed and relatively small area (1 × 10^6^ um^2^, > 1000 cells analyzed) took ∼2 min each. The sample fraction study was also carried out on the complete set of cases to assess its ability to differentiate NIFTP from invasive EFVPTC. It was observed through sensitivity and specificity study that increasing the sample fraction from 3 to 5 or 10 % did not increase the area under curve (AUC), shown in Supplementary Table 1A. ANOVA test revealed significant differences in both DIA parameters between NIFTP and invasive EFVPTC in all three sample fraction percentages tested (Supplementary Table 1B), again confirming 3% SURS of the tumor was sufficient to effectively differentiate NIFTP from invasive EFVPTC.

### DAB mean intensity at tumor sampled by hotspot nucleus best distinguished NIFTP from invasive EFVPTC

The PD-L1 expression parameters extracted from each subROI method were examined for its effectiveness to differentiate NIFTP from invasive EFVPTC. Mean intensity (continuous scale, average of pixel values from 255 to 0, light to dark) was superior to percent positivity (binary with each pixel coded as 1 or 0 for color intensity above or below a threshold) with lower *p*-values, larger AUC and smaller proportion of misclassified cases for all the subROI methods examined (Table 5). Under the criteria of low variance, high efficiency (less DIA processing time), and effectiveness in differentiating NIFTP from invasive EFVPTC (smaller *p*-value), the best performing fully-automated tumor sampling method was found to be Hotspot Nucleus (comparable *p*-value; however with ∼2–3 min. per slide in contrast to ∼4–5 min. for 3% SURS or Hotspot Nucleus and DAB), Table 6A. Comparing manual scores and DIA quantitation from each subROI method, it was observed Hotspot Nucleus had the highest concordance correlation coefficient value (0.66, 95% CI: 0.57, 0.73) (Table 6B).

**Table 5:** Clinical utility of tumor sampling methods

	Sens.	Spec.	PPV	NPV	AUC	Proportion Misclassified
	*NIFTP vs. EFVPTC*					
Manual subROI						
DAB Mean Intensity	75%	65%	66%	74%	0.77	30%
DAB Percent Positivity	71%	65%	65%	71%	0.75	33%
SURS 3%						
DAB Mean Intensity	67%	58%	58%	67%	0.71	38%
DAB Percent Positivity	67%	62%	60%	69%	0.70	36%
Hotspot – Nucleus						
DAB Mean Intensity	67%	66%	63%	70%	0.73	33%
DAB Percent Positivity	67%	62%	60%	69%	0.73	36%
Hotspot – Nucleus and DAB						
DAB Mean Intensity	67%	60%	59%	68%	0.70	37%
DAB Percent Positivity	67%	56%	57%	67%	0.67	39%
Manual Scoring	77%	64%	65%	76%	0.73	30%

Noninvasive follicular thyroid neoplasms with papillary-like nuclear features (NIFTP); encapsulated follicular variant of papillary thyroid carcinoma (EFVPTC); 3,3′-Diaminobenzidine (DAB); systematic uniform random sampling (SURS).

**Table 6:** subROI tumor sampling method evaluation

A								
*ANOVA Group Comparison*								
DIA Parameter	Group (I)	Group (J)	Comparison Groups	Mean Difference (I-J)	Sth.Error	Sig. (p)	95% CI	
							Lower Bound	Upper Bound
Percent Positivity	NIFTP	EFVPTC	Manual subROI	–24^*^	5.0	**<0.001**	–37	–11
			SURS 3%	–20^*^	5.6	**0.002**	–35	–6
			HotSpot-Nu	–22^*^	5.3	**<0.001**	–36	–8
			HotSpot-NuDAB	–15^*^	4.8	**0.009**	–28	–3
Mean Intensity	NIFTP	EFVPTC	Manual subROI	31^*^	6.3	**<0.001**	14	47
			SURS 3%	28^*^	7.1	**0.001**	10	47
			HotSpot-Nu	29^*^	6.7	**<0.001**	12	47
			HotSpot-NuDAB	23^*^	5.9	**0.001**	8	38

B					
Concordance Measurement					
Tumor Sampling Method	Covariance	Correlation Coefficient	Concordance Correlation Coefficient (Rc)	Rc 95% CI	
				Lower Bound	Upper Bound
Manual subROI	2.6	0.80	0.69	0.61	0.76
SURS 3%	2.4	0.71	0.64	0.55	0.72
HotSpot - Nu	2.5	0.75	0.66	0.57	0.73
HotSpot - NuDAB	2.2	0.75	0.65	0.56	0.72

^*^The mean difference is considered significant when *p* < 0.05.

>1000 viable tumor cells per case were evaluated.

Region of interest (ROI); Digital image analysis (DIA); programmed death-ligand 1 (PD-L1); noninvasive follicular thyroid neoplasms with papillary-like nuclear features (NIFTP); encapsulated follicular variant of papillary thyroid carcinoma (EFVPTC); systematic uniform random sampling (SURS); 3,3′-Diaminobenzidine (DAB)

## DISCUSSION

Distinguishing between invasive EFVPTC and NIFTP is clinically important and has gained increasing recognition. Given the wealth of insights that can be drawn from sectioned tissues, precise assessment of the protein biomarker expression could yield crucial ancillary diagnostic or prognostic information that have implications for personalized medicine and guiding treatment decisions [33].

Variability between expert assessments of IHC-stained tissue was reported to be high, reflecting the unevenness in histopathologists availability, difference in the level of training, and human factors (interobserver variability) [34–36]. Intratumor heterogeneity further negatively impacts pathologist concordance rates [37]. Nevertheless, surgeons, ENT specialists, pathologists and endocrinologists benefit from more precise diagnostic and prognostic evaluation of their patients’ thyroid tumors when protein biomarkers are assessed accurately [38]. Digital image analysis eliminates interobserver variability. A standardized and reproducible digital IHC protein biomarker assessment tool could offer means to efficiently and objectively quantitate the stain signal.

In this study, we developed and implemented a DIA protein biomarker IHC staining assessment algorithm in thyroid tissue using a histomorphological approach for PD-L1 expression. The ThyApp first processed the image as geometrical tissue components (colloid and stroma), and then applied operators to specifically select a spatially relevant element (the follicular cell). The software then performed quantitation and thresholding in this specified region. It consistently segmented thyroid variants with diverse histomorphology, extracted parameters reflecting stain variability, and effectively differentiated invasive EFVPTC from NIFTP and benign nodules by accurately quantitating PD-L1 expression in the follicular cells. The PD-L1 expression was significantly increased in EFVPTC with invasion comparing to NIFTP subgroup. Furthermore, the low PD-L1 expression in NIFTP comparable to benign nodules supports the concept that NIFTP are low risk thyroid cancers. However, we observed NIFTP PD-L1 expression spectrum distribution was positively skewed with a long tail and noted a few NIFTP cases with high PD-L1 expression scores. The close oncogenetic similarity and expression distribution overlap between the two encapsulated subgroups could contribute to the low sensitivity and specificity reported. Cho *et al.* indicated in a retrospective study that 3% of NIFTP had lymph node metastasis [39] and Parente *et al.* reported 6% of NIFTP showed evidence of malignant behavior [40]. These reinforcing the importance of detecting among NIFTP cases that could justify a possible concern for a carcinoma *in situ* risk or undetected lymphocytic infiltration and the need for cautious follow-up monitoring. We are cognizant that the limited follow up period (1.5–5 years) did not permit a clear statement regarding the relationship of the observed high PD-L1 expression in some NIFTP and their aggressiveness and/or recurrence. Nevertheless, the differential expression of PD-L1 in the different subgroups was significant and with further studies may support its future use in clinical management as an ancillary aid to a histopathologist report [14]. With continuing observations on the DIA test and its long term follow up on clinical outcomes, it will be possible using machine learning methods to improve the sensitivity and specificity of this test in the future. Cases of encapsulated follicular lesions/neoplasms without evidence of invasion and with coexisting lymphocytic infiltration were classified as a separate LT subgroup since the presence of mononuclear cell could elevate PD-L1 expression. This LT subgroup showed significant overexpression of PD-L1 suggesting high PD-L1 expression in thyroid tumors with coexisting chronic lymphocytic or Hashimoto's thyroiditis need to be interpreted with caution to avoid overdiagnosis and overtreatment of benign thyroiditis lesions [41, 42].

The ThyApp was performed in five circular subROI regions distributed pentatonically, sampling the tumor tissue. Each subROI (2 × 10^5^ um^2^) analyzed a minimum of 1000 viable tumor cells. The circular subROI was chosen (over square) for best conformability to the irregular tumor tissue delineated. The size of the subROI has shown in literature to impact DIA parameter output [43]. It was optimized not to be smaller as it reduced the sensitivity from small sampling (e.g. intratumor heterogeneous), yet not to be larger as it claimed more DIA processing time and decreased specificity of feature studied.

From the preliminary results of this patient cohort, both DIA parameters extracted for PD-L1 expression, DAB percent positivity and mean intensity, showed capability to differentiate invasive EFVPTC from NIFTP and benign nodules. In addition, higher significance was observed with mean intensity suggesting a quantitative parameter that harbors information about stain intensity variation in a continuous scale may be more precise than one that contains only semi-quantitative information (stain intensity transformed into binary code above or below a threshold). In extension to the ThyApp developed, here we also report the first tumor sampling methods evaluation study for DIA thyroid tissue. Upon user delineation of the tumor tissue, ThyApp was conducted in subROIs selected based on: expert guided manual subROI selection that relied heavily on the individual's knowledge and experience and three fully-automatic tumor sampling methods (SURS, Hotspot Nucleus, and Hotspot Nucleus and DAB). Based on the effectiveness, reproducibly, and efficiently to distinguish invasive EFVPTC from NIFTP, in this patient cohort, Hotspot Nucleus outperformed SURS and Hotspot Nucleus and DAB. In addition, it showed the highest concordance correlation coefficient to manual scoring suggesting the Hotspot Nucleus offered a simulated pathologist approach to focus on target areas where tumor cells were evaluated. Though Hotspot Nucleus and manual scoring showed similar AUC and proportion of misclassified cases, DIA is advantageous in providing reproducible data. The successful implementation of an objective and fully automatic DIA algorithm that reliably quantitates thyroid protein biomarker expression could be added to the pathologist's report.

No training set is required for the ThyApp used in this study. Global thresholding values used for segmentation were selected gearing the App to be less sensitive to background staining variation across image. Consistent preservation and staining processes applied to tissue provides reliable information. The assumption holds relatively true in patient cohort where specimens were processed and stained in the same institution and facility; however, as specimen preparation becomes dynamic (i.e. when unstained slides are obtained from different regions [44, 45]; sectioned tissues are stained at different facilities [46–48]; stained slides are scanned using different scanners [49]), the degree of stain variation due to processing on the obtained whole slide images increase [50]. An App with pre-specified thresholding values has a preset tolerance to variability. To meet clinical demands, evolving and equipping ThyApp to be more robust and resistant to IHC staining and scanning variations could be favorable. Unsupervised segmentation and automatic thresholding techniques utilizing advanced mathematical modeling (i.e. K-means clustering [51]) have the potential to mitigate the risks associated with the slide-to-slide IHC stain variation. To fully validate ThyApp's clinical utility, multicenter cohorts with subgroups classified by independent pathologists will be the subject of future studies. In addition, extracted feature reporting modalities could be investigated, including ordinal categorical scores and weighted score (i.e. H-score, assessing the immunoreactivity by a formula placing higher weight in strongly stained population and lesser weight to moderately and weakly stained nuclei). Considering lightly stained areas have higher potential to be noise, unspecific staining, or baseline protein expression, a smaller weight designated to this group of cells may be appropriate for assessing protein biomarker for tumor aggressiveness or malignant potential.

On the basis of this preliminary study and evaluation on the efficacy of DIA for IHC assessment in thyroid tissue, we report here a DIA method to quantitate protein expression using a histomorphological approach. The fully automatic DIA algorithm developed objectively quantitated PD-L1 expression in encapsulated thyroid neoplasms, distinguished invasive EFVPTC from NIFTP and benign nodules, and outperformed manual scoring in reproducibility and higher efficiency. The addition of digitalization of the glass slides and later computer assisted analysis could strengthen the argument supporting the justified cost required to perform DIA and further assist pathologists in diagnostic accuracy and subsequent personalized clinical management. Furthermore, the DIA method developed here specifying ROI for IHC quantitation by isolating the epithelium could also have potential applications for similar DIA in other glandular tissues, be it endocrine or exocrine, normal reactive or neoplastic types.

## MATERIALS AND METHODS

### Patients, specimens and immunohistochemistry

The retrospective study was approved by Research Ethics Board (REB #07-0212-E) of Sinai Heath System, Mount Sinai Hospital (MSH) in Toronto, Canada. From a total of 1859 cases of surgically removed thyroid nodules at MSH from Jan 2010 to May 2015, 153 cases were selected based on analysis of clinical charts, surgical pathology reports and histologic slides. Inclusion criteria for the study include: the presence of a recorded chart with the gender and age of the patient at the time of diagnosis, clinical history such as recurrence episodes, size of the nodule (more than 1 cm), focality (mono), and surgical pathology diagnosis of adenomatous nodules or EFVPTC as well as the existence of enough biological material in the paraffin blocks. It has been shown in the literature that NIFTP and EFVPTC share the same types of mutation [52] and NIFTP represents an indolent counterpart of EFVPTC. This study was performed using the similar patient cohorts with minor variation and inclusion/exclusion criteria for each subgroup studied as previously described in detail in our previous reports [13]. Archived formalin-fixed paraffin-embedded (FFPE) tissue blocks from the selected 153 thyroid nodules were retrieved from the MSH pathology tumor bank. Hematoxylin and eosin stained tissue sections were reviewed by two pathologists (CM, OP) and classified into four specific diagnostic subgroups: NIFTP (48 cases) and EFVPTC (44 cases), benign nodules (26 cases) and encapsulated follicular lesions/neoplasms with coexisting advanced lymphocytic or Hashimoto's type lymphocytic thyroiditis (LT) (35 cases) [33, 53–55]. Briefly, the benign cases included adenomatous hyperplastic nodules and adenomas and were identified based on the presence of clear demarcation and/or encapsulation, follicular architecture and nuclei without PTC features [56]. The NIFTP subgroup included cases with well-demarcated and/or encapsulated lesions with follicular architecture, PTC-type nuclei and absence of capsular or vascular invasion. EFVPTC were diagnosed based on PTC nuclear features, an almost totally follicular architecture [56] and tumor invasion into capsule or extension into adjacent thyroid tissue and/or presence of lymphovascular invasion [55]. The cases with lymphocytic thyroiditis were separated out to remove the potential influence of lymphocytic inflammation on PD-L1 expression in these types of tumors. Previously such influence had been reported [11]. Lymphocytic thyroiditis was diagnosed based on lymphocytic infiltration with formation germinal centers and patchy disruption and collapse of thyroid follicles. For cases with Hashimoto's thyroiditis increased interstitial connective tissue was additionally observed with foci of Hurthle cell metaplasia [56]. The cases with coexistence of advanced lymphocytic or Hashimoto's thyroiditis were encapsulated follicular lesions/neoplasms which did not satisfy inclusion criteria for NIFTP and had no evidence of capsular or vascular invasion.

Immunohistochemistry staining procedures were performed and described in detail by Fu *et al.* [13]. Briefly, 4 μm FFPE tissue sections were deparaffinized and rehydrated. Antigen retrieval was performed followed by the application of background punisher. The sections were incubated overnight at 4°C with the monoclonal rabbit anti-PD-L1 antibody at 1:100 dilution (E1L3N, Cell Signaling Technology, Inc. Danvers, MA). The VECTASTAIN rapid protocol (Vector Labs, Burlington, ON, Canada) with Diaminobenzidine (DAB) as the chromogen was performed. Hematoxylin was used as counterstain. Typical examples of PTC and histiocytes were used as positive control and internal controls, respectively. Isotype specific IgG in place of primary antibody was used as a negative control.

### Manual scoring of immunostaining

Immunostaining scores were based on the percentage positivity and staining intensity as described previously [11]. Percentage positive scores were assigned according to the following scale: 0 = < 10%; 1 = > 10–30%; 2 = > 31– 50%; 3 = > 51–70%; and 4 = > 71%. Staining intensity was scored semi-quantitatively as follows: 0 (none); 1 (mild); 2 (moderate) and 3 (intense) [5]. A total score for each cytoplasmic and membranous staining was then obtained (ranging from 0 to 7) by adding the percentage positivity scores and intensity scores for each section. The IHC scoring was blinded from the histopathology report and was performed by two evaluators independently and used for subsequent analyses. The final score was given by averaging the two total scores from the two evaluators with equal weights. The final score was given using following formula [13]: [(percentage score 1 + intensity score 1) + (percentage score 2 + intensity score 2)]/2.

### Whole slide image capture

Nano Zoomer 2.0 RS (Hamamatsu Photonics K.K., Hamamatsu, Japan), whole slide images were captured with a 20X objective (Achroplan 40X/0.65 ∞/0.17) with a Coolsnap digital camera (Coolsnap, RS Photometrics, Tucson, AZ). Digitalized images have a resolution of 1392 × 1040 pixels with RGB 24 True Color format and were saved in NDP format. The same range of illumination values was used to ensure the greatest reproducibility.

### Digital image analysis

ThyApp, a systematic image processing operation algorithm was developed in our lab using the Visiopharm integrator system for Windows 7, version 5.0.1.1122 (Visiopharm A/S, Denmark). DIA analysis were carried out on a standard off-the-shelf desktop computer.

The Visiopharm integrator system software runs on an image processing pipeline. Specific add-on modules implemented are listed below. In preprocessing, median filter were applied to perform background subtraction and noise reduction. Color deconvolution filters (Hematoxylin and DAB) were applied to separate the color space. Next image pixels were segmented, defining different tissue subcomponents (colloid and non-colloid). The elementary image is no longer individual pixels but connected sets of pixels belonging to the same group. In the post processing step, the segmented layer labels were refined based on the morphological and contextual of the surrounding elements. The cell to cell spatial relationship that drives the structural organization of the thyroid tissue was explored. Interface between parafollicular space and colloid was specified as the follicular cells for IHC staining quantitation. Nuclei of these cells were excluded for the subcellular staining assessment. Second thresholding on the pixel intensity in the IHC quantitation area was performed. Lastly, features were extracted and exported as DIA parameter values including percentage of positively IHC labelled cells (area of DAB intensity color value below a threshold gate to total area IHC quantified, 0–100%) and digital immunostaining intensity in the subROIs quantified (color value 0–255, dark to light brown). Both are continuous numerical values given equal weight to every pixel analyzed.

### Tumor sampling methods

The WSI was DIA analyzed using a multi-scale feature extraction approach that mimics the human eye when examining a slide. First, an operator delineated the tumor tissue exclusion of CIS, ink artifacts, tumor satellites and all tumor areas with intense inflammation, fibrosis, necrosis or poor fixation and termed it ROI. Next, areas within the ROI, termed subROIs, where the software performs the quantitation at high resolution were selected. One semi-manual and three fully automatic subROI selection methods were evaluated. In semi-manual, subROIs for DIA were manually selected by a histopathologist. Three fully automatic subROI selection methods: Systematic Uniform Random Sampling (SURS) (a specified sample fraction of tumor); hotspot nucleus (Heatmap algorithm selects subROIs at highest tumor cell nuclei density); hotspot nucleus and DAB (Heatmap algorithm selects subROIs at highest tumor cell nuclei density and DAB positivity) were individually implemented.

### Statistical analysis

The data collected in a database designed for this study were analyzed using the SPSS version 20 (SPSS Inc., Chicago, IL, USA) program. A descriptive analysis of the population was performed. The qualitative variables were described with frequencies, and the quantitative variables were described with central tendency measures: means, standard error and dispersion standard deviation, range to 95%. A bivariate analysis was performed using independent samples *t*-test for quantitative variables. PD-L1 scores were compared among the different diagnostic groups using one-way ANOVA test. The results were considered significant when the obtained values were *p* < 0.05. Hypothesis tests were 2-sided. For measurement of linear relationship between manual and DIA scoring of PD-L1 expression, Pearson covariance and correlation coefficient were computed. In addition, for measurement of concordance, Lin's correlation coefficient was computed. Shapiro-Wilk test was performed to determine normality of the scores in each subgroup.

## SUPPLEMENTARY MATERIALS FIGURE AND TABLE


