# Supplementary material for: Programmed death-ligand 1 expression by digital image analysis advances thyroid cancer diagnosis among encapsulated follicular lesions
Source: Oncotarget. 2018 Apr 13;9(28):19767–82. doi: 10.18632/oncotarget.24833 (PMC5929424; doi:10.18632/oncotarget.24833)
Supplement: Supplementary file 1 [file oncotarget-09-19767-s001.pdf]

Programmed death-ligand 1 expression by digital image analysis advances thyroid cancer diagnosis among encapsulated follicular lesions

SUPPLEMENTARY MATERIALS

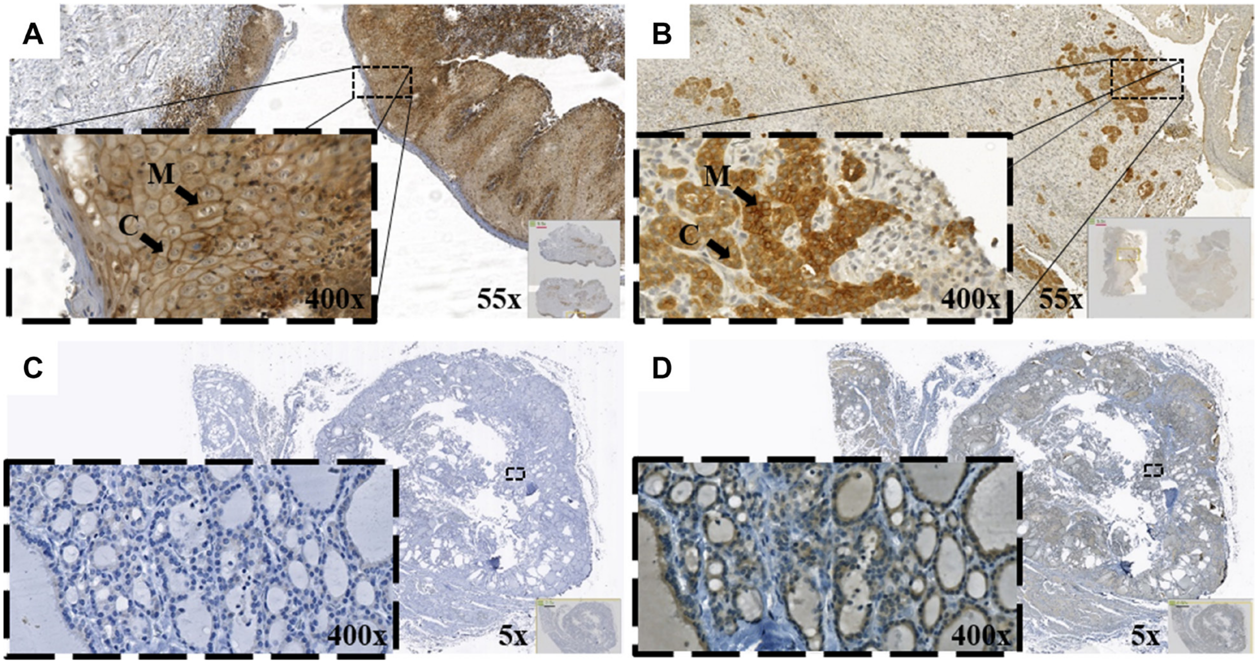

Supplementary Figure 1: Immunohistochemical staining controls for PD-Ll expression. (**A**) Positive tissue type control, an oral cancer tissue section showed PD-LI membranous and cytoplasmic staining. (**B**) Positive tissue type control, an aggressive anaplastic thyroid cancer section showed strong membranous and cytoplasmic staining. (**C**) Isotype negative control, an encapsulated FVPTC section incubated with isotype specific IgG in place of the anti-PD-LI antibody did not show detectable immunostaining. (**D**) Positive batch control, the same encapsulated FVPTC case stained with anti-PD-LI antibody showed moderate immunostaining. Image magnification are ×55 for (A and B), ×5 for (C and D), and ×400 for (A–D) insets. Programmed death-ligand I (PD-LI); Follicular variant of papillary thyroid carcinoma (FVPTC).

Supplementary Table 1: SURS tumor sample fraction study

A

	**Sens.**	**Spec.**	**PPV**	**NPV**	**AUC**	**Proportion Misclassified**
**SURS**	*NIFTP vs. EFVPTC*					
3%						
DAB Mean Intensity	67%	58%	58%	67%	0.71	38%
DAB Percent Positivity	67%	62%	60%	69%	0.70	36%
5%						
DAB Mean Intensity	67%	62%	60%	69%	0.70	36%
DAB Percent Positivity	67%	58%	58%	67%	0.69	38%
10%						
DAB Mean Intensity	67%	58%	58%	67%	0.70	38%
DAB Percent Positivity	67%	56%	57%	67%	0.70	39%

B

**DIA Parameter**	**Group (I)**	**Group (J)**	**Comparison Groups**	**Mean Difference (I-J)**	**Std. Error**	**Sig.**	**95% CI**	
							**Lower Bound**	**Upper Bound**
Percent Positivity	NIFTP	EFVPTC	3 %	–20*	5.6	**0.002**	–35	–6
			5 %	–20*	5.3	**0.002**	–34	–6
			10 %	–20*	5.3	**0.001**	–34	–6
Mean Intensity	NIFTP	EFVPTC	3 %	28*	7.1	**0.001**	10	47
			5 %	27*	6.8	**0.001**	10	45
			10 %	28*	6.7	**<0.001**	10	45

*The mean difference is significant at the 0.05 level.

>1000 viable tumor cells per case were evaluated.

Systematic uniform random sampling (SURS); noninvasive follicular thyroid neoplasms with papillary-like nuclear features (NIFTP); encapsulated follicular variant of papillary thyroid carcinoma (EFVPTC); 3,3′-Diaminobenzidine (DAB).
